# Emotional and Motivational Pain Processing: Current State of Knowledge and Perspectives in Translational Research

**DOI:** 10.1155/2018/5457870

**Published:** 2018-07-18

**Authors:** Susanne Becker, Edita Navratilova, Frauke Nees, Stefaan Van Damme

**Affiliations:** ^1^Department of Cognitive and Clinical Neuroscience, Central Institute of Mental Health, Medical Faculty Mannheim, Heidelberg University, Mannheim, Germany; ^2^Department of Pharmacology, University of Arizona, Tucson, AZ, USA; ^3^Department of Experimental-Clinical and Health Psychology, Ghent University, Ghent, Belgium

## Abstract

Pain elicits fear and anxiety and promotes escape, avoidance, and adaptive behaviors that are essential for survival. When pain persists, motivational priority and attention shift to pain-related information. Such a shift often results in impaired functionality, leading to maladaptive pain-related fear and anxiety and escape and avoidance behaviors. Neuroimaging studies in chronic pain patients have established that brain activity, especially in cortical and mesolimbic regions, is different from activity observed during acute pain in control subjects. In this review, we discuss the psychophysiological and neuronal factors that may be associated with the transition to chronic pain. We review information from human studies on neural circuits involved in emotional and motivational pain processing and how these circuits are altered in chronic pain conditions. We then highlight findings from animal research that can increase our understanding of the molecular and cellular mechanisms underlying emotional-motivational pain processing in the brain. Finally, we discuss how translational approaches incorporating results from both human and animal investigations may aid in accelerating the discovery of therapies.

## 1. A Shift to Negative Emotional-Motivational Processing in Chronic Pain

Pain is much more than the conscious perception of a sensory event. It is aversive and inseparably linked to emotion as reflected in the generally accepted definition of pain as “an unpleasant sensory and emotional experience associated with actual or potential tissue damage, or described in terms of such damage” [1]. In chronic pain, a high comorbidity with affective disorders such as depression and anxiety has been reported [2]. It has been hypothesized that in chronic pain, a predominance of processing of negative emotional information might underlie emotional-motivational problems such as depression and anxiety. Research on pain has shifted in the last couple of decades from a strong focus on nociception toward a broader perspective that includes factors such as pain-relevant emotion and motivation that may contribute to the development and maintenance of chronic pain [3, 4].

A central feature of pain is that it is a strong stressor and motivator that can induce fear and anxiety and urges escape and avoidance responses. Acute pain is physiological by acting as an alarm system, thereby preventing further damage and promoting behavior directed toward healing. However, in chronic pain, fear, anxiety, and escape and avoidance responses become maladaptive, often resulting in impaired functionality, social withdrawal, anxiety, and depression [5]. The idea that chronic pain is related to a negative emotional-motivational shift is intriguing because it provides an explanation for dysfunctional processes related to reward, learning, goal regulation, and avoidance and approach behavior. However, investigating the mechanisms underlying a shift to negative emotional-motivational pain processing is challenging. Animal models allow important insights into neurobiological mechanisms; however, assessment of emotional-motivational aspects of pain is not comprehensive. In contrast, subjective experiences can be easily assessed in humans, but uncovering underlying neurobiological mechanism is more difficult. Translational approaches offer benefits linking these two domains.

In this review, we focus on emotional-motivational factors possibly underlying a shift towards negative processing in chronic pain, bringing together information from animal and human research. Reward processing, learning, goal regulation, and related pain avoidance behavior are such factors that seem to play a prominent role in pain and that are likely altered in the proposed shift. Possible synergies of translational research are highlighted, along with approaches on how preclinical research can be informed by human studies and vice versa.

## 2. Human Studies to Investigate an Emotional-Motivational Shift Towards Negative Affective States in Chronic Pain

Human behavior is directed by goals in a broad array of domains (e.g., social, work-related, health). Thus, understanding an individual's response to acute and ongoing pain requires thorough examination of the motivational dynamics between this pain and other demands. Self-regulation theories, describing the processes by which one attempts to gain control over behavior, thoughts, feelings, desires, and actions in the service of goal attainment [6, 7] provide useful insights into a range of phenomena including avoidance behavior and attentional processes [8]. An important aspect of self-regulation is how people modulate negative states, including pain, in the process of goal pursuit. Ongoing pain is often perceived as a barrier to the pursuit of valued goals [9]. The presence of ongoing pain results in perceived or anticipated discrepancies between actual and desired behavioral outcomes, resulting in negative effect. In an early state, patients with persistent pain are often motivated to stay committed to earlier (prepain) life goals and performance standards [10]. This is reflected by task persistence despite pain, which requires cognitive shielding and effort mobilization [11]. For example, attention will be focused on goal-relevant information, whereas distracting pain information will be inhibited [12]. In case of repeated goal failure, however, a motivational shift towards pain-related goals might occur, which often becomes a very dominant goal in patients' goal hierarchy. Individuals then prioritize effort towards goals such as getting rid of or acquiring control over the ongoing pain problem, which is often reflected by increased problem-solving attempts and avoidance behavior [13], which are often maladaptive in ongoing and chronic pain. Such motivational shift is typically associated with a narrowed focus of attention toward pain-related information, increased accessibility of negative information, and worrying [10, 14]. Repeated failure of problem-solving attempts and attainment of valued goals might, at some point, start inducing feelings of helplessness and hopelessness and associated states of depression and anhedonia. According to Brandtstädter and Rothermund [11], such (temporary) states may be adaptive, because they urge reappraisal of the current situation and disengagement from inefficient effort mobilization to unattainable goals. Goal disengagement is reflected by devaluation of blocked goals, acceptance, and a shift of attention from narrowly focused and top-down towards more holistic, broad-based, and bottom-up, allowing activation of alternative goals that may be more feasible and less affected by pain.

In addition to goal regulation, self-regulation theories consist of many factors, such as reward processing, learning, and approach and avoidance. All these factors, in themselves, have suggested mechanisms likely involved in an emotional-motivational shift in chronic pain. For example, reward processing has been dissected into dissociable components of wanting, that is, the motivation to obtain a reward, and liking, that is, the hedonic experience of such a reward [15]. Both components can be differentially affected by pain [16, 17]. Pain has been reported to increase wanting while leaving liking unaltered [5, 18]. This mismatch may reflect a drive to compensate for a negative emotion induced by pain, but being unsuccessful to increase pleasure. In chronic pain, a similar mismatch could represent unsuccessful coping attempts, and the lack of increased pleasure following “trying harder” could potentially lead to phenomena like learned helplessness [16]. Learning comprises several processes that are mechanistically very different in promoting behavior and/or perception. For example, operant learning results from the consequences of a behavior where the desired consequences lead to an increase in the probability that preceding behavior will be shown again (i.e., reward), while undesired consequences lead to a decrease of this probability (i.e., punishment) [19]. In contrast, respondent or Pavlovian learning is the process, by which a formerly neutral stimulus comes to elicit a response that is similar as a response elicited by a naturally meaningful stimulus. Pain is a meaningful stimulus, eliciting, among other reactions, fear. If this pain is repeatedly coupled to a neutral stimulus, for example, a specific situation or movement (which does not cause the pain directly), this conditioned stimulus (CS) can then elicit fear [20]. Approach and avoidance can be the results of such learning. For example, one can learn to avoid pain- or fear-inducing situations. Avoidance leads to particularly robust behavior. Because of avoidance, a person does not experience that a feared stimulus is no longer harmful or threating, potentially delaying recovery. Such processes are well known in pain [21, 22].

The theoretical framework presented here, providing insight in how people struggle to make sense of unwanted experiences and how they avoid, adapt, or alter the perceived causes of those experiences, offers an excellent starting point to investigate emotional-motivational shifts in chronic pain at behavioral and psychobiological levels in humans. These processes are related to subjective experience and higher cognitive function, rendering human research an essential part in advancing knowledge on the emotional-motivational shift in chronic pain.

### 2.1. Research in Healthy Volunteers

#### 2.1.1. Reward Processing

Pain and reward or pleasure has been suggested to be the two ends of a continuum, with emotion as a common currency that allows the comparison between stimuli [23]. This assumption suggests that pain and reward/pleasure affect each other. Indeed, pleasure and appetitive motivation induced by pleasant odors, pictures, or music as well as monetary rewards inhibit acute pain [24–27]. An interesting case of reward is pain relief. Pain relief is strongly sought-after when in pain and can become an all-dominant goal in chronic pain. Relief from an aversive state is generally associated with rewarding experiences. For example, food tastes better when one is hungry [28, 29], and the pleasure of pain relief is known to almost everybody. In line with the results on effects of pleasure and (monetary) reward on pain perception, pain relief as reward induces endogenous pain inhibition over and above the physical pain reduction [30].

The idea of a hedonic continuum with pain and reward/pleasure at its ends is supported by the observation that brain processing of pain and reward widely overlap [31]. But, only few studies so far investigated the mediating mechanisms between interactions of pain and reward. One study demonstrated that dopamine mediates pain inhibition induced by reward [27]. Dopamine mediated not only the pain-inhibiting effects of reward, but also the pain-facilitative effects of punishment suggesting that dopamine mediates motivated behavior triggered by stimuli of positive and negative valence [32]. With respect to endogenous opioid activation, it has been observed that responses in the periaqueductal gray and ventral striatum to rewarding stimuli at the age of 14 years predicted the presence of pain complaints in adolescents two years later, with much stronger effects for carrier of the T-allele of the rs563649 polymorphism of the human mu-opioid receptor (OPRM1) gene [33]. Carriers of the T-allele of this polymorphism shown increased responsiveness to a number of noxious stimuli, including ischemic, mechanical, and thermal stimuli applied to various anatomical sites [34]. At the circuit level, the orbitofrontal cortex mediates reward-induced pain inhibition through altered functional connectivity with the rostral anterior insula, anterior-dorsal cingulate cortex, and primary somatosensory cortex [35]. In line with these results, Talmi et al. [36] showed that increased activation in the insula was associated with prioritizing pain avoidance over obtaining a monetary reward, and this increased activation was related to increased activation in the orbitofrontal cortex. Pain relief (although not implemented as reward) is associated with increased activation in the nucleus accumbens as well as in the ACC [37–39]. The ACC has a high density of opioidergic receptors, leading to the hypothesis that opioidergic mechanisms in the ACC might be related to the endogenous pain inhibition related to rewarding pain relief, which is supported by preclinical studies (see below). In humans, this was demonstrated by positron emission tomography (PET) showing an association of opioidergic receptor activation in the ACC with perceived reductions in pain induced by placebo interventions [40–42].

#### 2.1.2. Learning

Learning processes can affect the pain experience via different routes. One possibility is through operant learning, that is, learning based on reward, affecting the perception of pain. Few studies investigated this route by using, for example, verbal reward in terms of praise to increase or decrease participants' ratings of experimental pain [43, 44]. However, it is conceivable that in those studies, only participants' rating behavior was changed (as this was what was reinforced), but not necessarily the perception of pain. But, by operant learning, also pain-related evoked brain potentials [45] and facial pain expression [46] can be increased or decreased. Instead of using extrinsic reinforcement, such as verbal reinforcement or smileys, pain relief can be used as intrinsic reinforcement, that is, within the pain system. As such, rewarding pain relief appears particularly well suited to induce operant learning processes, which has been shown in several studies, confirming that (partial) pain relief can lead to learned changes in pain perception in humans [47–49] and in animals (e.g., [50]; see Section 3 below).

A different route is fear learning that affects pain processing. Learned fear of pain is related to facilitated sensitization and decreased thresholds for pain [51–53], interference with habituation to repeated pain stimuli [54], enhanced somatosensory processing at pain-relevant body locations [55, 56], and impaired acuity of perceptual discrimination [57]. Pain-related fear conditioning has also been shown to induce increased tension in muscle responses [58].

#### 2.1.3. Goal-Regulation, Approach, and Avoidance

The effect of motivational context on pain processing has been the topic of increasing experimental research endeavors. For instance, it has been demonstrated that experimentally inducing the goal of pain avoidance in healthy volunteers significantly enhanced existing attentional biases to visual cues signaling imminent pain [59]. Durnez and Van Damme (2015) [60] found that experimentally induced expectation of pain on a specific body location enhanced somatosensory processing at that body location relative to other locations in a Tactile Change Detection (TCD) task, especially in participants who were motivated to actively avoid painful stimulation by a specified motor action. Durnez and Van Damme [61] further demonstrated that using similar experimental manipulation of motivation, participants actively attempting to avoid pain showed prioritization of somatosensory stimuli over visual stimuli in a bimodal Temporal Order Judgment (TOJ) task. It has also been found in an experimental study with healthy volunteers that after losing control over pain administration, persistent attempts to regain control were associated with more fear of pain and performance costs on a secondary cognitive task, suggesting narrow attentional focus on pain [62]. All this experimental work suggests that alterations in goal priorities, such as a strong motivational focus on pain control or avoidance, might substantially enhance attentional focus on pain.

Some experimental work studying the effects of competition between pain-related goals and nonpain goals is available. It has been shown that concurrent pursuit of a salient goal significantly reduced attentional bias towards visual cues signaling impending experimental pain [63]. Furthermore, Karsdorp et al. [64] used a dot probe paradigm to assess attentional bias in which stimuli predicting experimental pain were put in competition with stimuli associated with a temporary nonpain goal. They found preferential attending to goal-related information over pain-related information, especially in those participants high in self-reported trait attentional control. Furthermore, increasing the motivational salience of a distraction task during cold pressor pain enhanced distraction effectiveness, particularly in participants scoring high on catastrophic thinking about pain [65]. There is also increasing evidence that induction of a nonpain goal may counteract pain-related avoidance behavior. In a study by Van Damme et al. [66], healthy volunteers were presented trials of two different tasks, of which one could result in the administration of a painful stimulus, and they were free to perform or not these trials. In half of the sample, a competing goal was induced by instructing participants that they could win monetary rewards by performing the pain task. The findings showed that this competing goal resulted in less avoidance of the painful task and in a significant reduction of the association between fear of the pain stimulus and avoidance of the painful task. Similar findings were reported in a study by Claes et al. [67]. Using a voluntary joystick paradigm, they conditioned one movement to be pain-inducing and another movement to be safe. In half of the sample, the pain-inducing movement was also associated with obtaining a financial reward. In those participants, the pain-related movement was performed more frequently, and the typical slowing of movements signaling pain was attenuated, although pain-related fear remained unaltered. These findings were replicated in a study by Claes et al. [68]. They used the same procedure, but additionally categorized participants based on their self-reported goal priority (avoiding pain versus seeking reward) and found that the effect of the concurrent goal on pain avoidance behavior was most pronounced in those participants who prioritized reward seeking, and less pronounced in those participants prioritizing pain avoidance. This is also in line with another study which experimentally manipulated goal pursuit during the performance of cognitive tasks while being exposed to pain and found that participants persisted longer in an achievement relative to a hedonic (avoiding pain) goal context [69].

### 2.2. Research in Chronic Pain Patients

#### 2.2.1. Reward Processing

Although studies are still scarce, research suggests changes in brain functions in individuals with chronic pain [3, 70]. Impairment of reward-related decision-making has been reported in patients with chronic back pain and complex regional pain syndrome (CRPS) [71]. Studies have shown that chronic pain is likely to be accompanied by a hypodopaminergic state [32]. A decrease in D2-receptor binding [72–75] and presynaptic activity [76, 77] in the striatum has been observed at rest and after painful stimulation in humans using PET. This might also play a critical role in chronic pain. Further, altered responses in the nucleus accumbens to the cessation of a painful stimulus were found in patients with chronic pain versus healthy controls using fMRI [37]. Moreover, variations of the activity in the nucleus accumbens during reward processing significantly predicted anhedonia, that is, the inability to perceive pleasure, which has been suggested to be associated with chronic pain in some patients independent of depression [78, 79]. In addition, chronic pain patients have been shown to exhibit less robust activation in brain regions associated with affective and cognitive pain-modulatory processes, specifically the ventral tegmental area, to the anticipation of pain (pain onset) and the anticipation of pain relief (pain offset) [80].

#### 2.2.2. Learning

The few available data on respondent conditioning in patients with chronic pain suggest that pain responses can be easily conditioned on multiple levels of the nervous system and can lead to enhanced pain perception and maladaptive brain changes, and that chronic pain patients acquire conditioned fear responses faster and extinguish them more slowly [58, 81, 82]. In patients with chronic low back pain compared to healthy individuals, a positive correlation of brain responses in the amygdala and insula and their connectivity with conditioned fear of movement has been observed [83]. Moreover, in patients with irritable bowel syndrome (IBS) compared to healthy individuals, increased cerebellar responses to both pain-associated and safety conditioned stimuli (CSs) during fear learning have been found. With respect to fear extinction, chronic pain may be characterized by impaired extinction of pain-related fear generalization [84] and enhanced reactivations of extinguished conditioned fear responses [85–87]. This may indicate a preservation of pain disability in chronic pain by persistent excessive protective behavior, which seems to be related to an individual's awareness of the association between the CS and the pain, which is the unconditioned stimulus [88, 89].

Moreover, along an operant conditioning model of pain [90], increases or decreases of pain perception can serve as implicit reinforcers: in this context, fibromyalgia patients without comorbid IBS did not learn pain habituation but showed increased sensitization compared to healthy controls, and those with comorbid IBS showed neither sensitization nor habituation learning [49]. Mechanisms of operant conditioning of sensitization or habituation may thus be differentially altered in chronic pain.

In addition, respondent and operant processes interact and may induce motivational biases forming habitual behaviors and a shift from more ventral to dorsal striatal circuits similar to addictive processes [91]. Thus, in chronic pain, fear of pain may then motivate behaviors that lead to pain relief, which is experienced as rewarding. However, this still needs to be tested in an experimental fashion. Moreover, brain changes during fear-conditioned predictions such as reduced PFC responses during extinction learning may hinder learning of new predictive stimulus properties. This includes those that are important to reduce pain and could thus contribute to the development and persistence of chronic pain, but also this still needs to be determined.

#### 2.2.3. Goal Regulation, Approach, and Avoidance

One of the most significant alterations resulting from chronic pain conditions is the disruption of appropriate and valued (goal-directed) behavior [92–94]. Changes in approach and avoidance tendencies are discussed as central components in the development and maintenance of chronic pain [95]. The competition between pleasure-related cues and pain can drive decisions and guide approach or avoidance behavior, which can already be observed at stages of acute pain. Pain-related fear needs to be considered within a motivational context to avoid or control pain with often competing existing goals [10, 13, 96–100]. Patients with chronic pain often experience difficulties in weighing the value of pain avoidance versus the withdrawal from valued activities [16, 101–103]. This would be especially important in the case when pain relief becomes more important than other rewards.

Correspondingly, it has been proposed that individuals with chronic pain are often stuck in (largely unsuccessful) attempts to get control over their pain [14] and that this makes them chronically vigilant to pain-related information [104]. A recent study indicated that individuals with chronic pain give indeed high priority to pain control goals and often see pain control as a necessary condition to attain other goals [105]. There is also accumulating evidence that chronic pain patients might be characterized by biased attention to pain-related information [106]. However, empirical research on the link between shifts in motivational priority and attentional processing is scarce [12]. One study, though, showed that a strong focus on problem-solving attempts towards pain was associated with more catastrophic thinking about pain and greater attention to pain [107].

## 3. Preclinical Investigations of Emotional-Motivational Pain Processing

Although most animal studies of pain rely on measurements of evoked mechanical and thermal responses, the need for evaluation of affective/motivational and cognitive aspects of pain and the underlying molecular, cellular, and circuit mechanisms is increasingly being recognized [108]. Complementary to human studies, animal investigations have demonstrated a critical role of corticolimbic circuitry in the processing of acute and chronic pain, which is involved in the evaluation of changing goals and competing motivations and facilitates decisions on avoidance, coping, and adaptive behavior. During acute pain states, corticolimbic circuitry promotes self-regulation of pain [109] through the engagement of descending pain-modulatory pathways or through the inhibition of the affective/emotional response to pain.

### 3.1. Animal Studies of Pain-Motivated Behavior

Mesolimbic reward/motivation circuitry, consisting of dopaminergic neurons in the ventral tegmental area and their projections to the nucleus accumbens in the striatum, is activated by rewards including food, drink, warmth, and so on and by drugs of abuse. Electrophysiological studies in animals, typically demonstrate inhibitions of midbrain dopamine neurons by aversive stimuli, consistent with the role of these neurons in reward coding [110–112]. However, a proportion of dopaminergic neurons can be excited by noxious or alerting stimuli [113–115]. In addition, some reward coding dopamine neurons were found to increase their activity at the offset of a noxious stimulus [116, 117], suggesting a “rebound” response to relief of pain. Therefore, mesolimbic neurons respond to alerting, aversive, and rewarding stimuli, including relief reward [118], and encode their salience and motivational value. These heterogeneous populations of neurons project to different brain regions in the striatum, prefrontal cortex, hippocampus, and amygdala to initiate specific motivated behavior and to promote learning.

Recently, measurements of dopamine transients in response to painful pinch of the rat's tail using fast scan cyclic voltammetry (FSCV) demonstrated increased dopamine release in the dorsal striatum and in the core of the nucleus accumbens [119], suggesting saliency coding. In contrast, dopamine levels were suppressed during the painful stimulus in the shell region of the nucleus accumbens and increased after pain offset [119, 120]. Behavioral studies in rats with sustained postsurgical, neuropathic, inflammatory, or bone cancer pain demonstrate that relief of pain with nonopioid interventions elicits conditioned place preference (CPP) only in injured but not sham-operated animals [50, 121–127]. These findings demonstrate that pairing of the pain-relieving treatment with a CPP chamber provides a learning experience and promotes motivation to seek pain relief. CPP is accompanied by release of dopamine in the NAc and blockade of dopamine receptors in the nucleus accumbens blocks CPP from pain relief [50]. These studies establish the role of mesolimbic dopamine signaling in pain and pain relief as alerting and emotional signals that promote learning and shape behavioral response.

### 3.2. Aversive Aspects of Pain

The prefrontal cortex (PFC) plays a major role in motivated behavior, decision-making, emotional processing, and cognition and is therefore critically involved during acute and chronic pain. The PFC provides top down control of the sensory and affective dimensions of pain and has functional connections with the mesolimbic dopamine circuitry, amygdala, and hippocampus. In rodents, PFC can be subdivided into the anterior cingulate (ACC), prelimbic (PL), and infralimbic (IL) cortices, each providing unique pain-modulatory functions. In the ACC, long-term potentiation (LTP) is believed to enhance pain responses and facilitate the interaction between chronic pain and anxiety [128]. Optogenetic stimulation of the inhibitory neural circuitry of the ACC leads to decreased neuronal activity in the ACC and reduction in pain behavior [129]. Pharmacological studies demonstrating CPP in rats with neuropathic pain following microinjection of morphine in the ACC indicate that these pain inhibitory circuits are likely the targets of exogenous opioid analgesics [130]. In addition, blockade of opioid ACC circuits prevented CPP elicited by nonopioid therapy, suggesting that endogenous opioid ACC circuits may be necessary for relief of pain aversiveness in general [130].

In the rodent PL, chronic neuropathic pain was shown to inhibit firing of pyramidal neurons as a result of feed forward inhibition mediated by GABAergic interneurons [131]. Consequently, optogenetic activation of parvalbumin-positive GABAergic neurons decreased pain responses and produced CPP, while optogenetic inhibition of these cells increased pain responses. Moreover, antinociceptive effects were also observed following selective activation of the projections from PL to NAc, suggesting regulatory role of the PL on mesolimbic dopamine circuitry and affective/motivational symptoms of pain [132].

The PFC also receives reciprocal inputs from the amygdala and the hippocampus, the brain regions essential for consolidation of emotional memories and fear conditioning. Synaptic plasticity in both regions has been observed in rodent models of neuropathic pain [133, 134]. Deactivation of the PFC through increased inputs from the basolateral amygdala has been demonstrated in a model of arthritic pain [135, 136]. Conversely, the PFC projects to and modulates the activity of a specific population of inhibitory cells in the amygdala, the intercalated cells, that control the function of amygdala pain output cells. However, more research needs to be done to unravel the precise neural circuitry that drives specific pain-related behavior.

Preclinical research therefore supports a critical role of the corticolimbic neural circuits involving endogenous dopamine and opioid neurotransmission in adaptive behavior, learning, and decision-making in the context of pain [137]. Corticolimbic circuits integrate pain-related experiences with other competing motivational goals, allow evaluation of the costs and benefits, and underlie selection of appropriate behavioral actions. The reported results in animals are therefore well in line with result in humans. The engagement of corticolimbic circuits in endogenous pain adaptive mechanisms also implies that diminished ability of these circuits to control emotional pain processes may underlie the transition to chronic pain [138].

### 3.3. Aberrant Corticolimbic Circuitry and Transition to Chronic Pain

Animal pain studies are typically conducted at early times following injury (less than one month) and cannot be classified as truly chronic and analogous to chronic pain conditions in patients. However, several recent studies have extended this time period in order to identify neural mechanisms that occur late after the initial insult and may therefore represent mechanisms underlying the shift from sensory to emotional pain processing. Hubbard et al. [139] and Seminowicz et al. [140] have investigated pain behavior and brain changes during a 4-month period following nerve injury in rats. At this late time point, they observed the emergence of anxiety behavior that coincided with the occurrence of volumetric changes in the PFC of injured rats. It is likely that molecular and cellular modifications in the brain precede these larger-scale volumetric changes and the emergence of comorbid behaviors such as anxiety, depression, and cognitive impairments. Indeed, expression of genes encoding dopamine and kappa opioid receptors in the rat nucleus accumbens was reduced 28 days but not 5 days following nerve injury [141]. Coincidently with these molecular changes, altered functional connectivity between the NAc and dorsal striatum and cortex was also observed only at the later time point. Moreover, fMRI BOLD activity in the NAc and prefrontal areas was associated with tactile allodynia on day 28 after nerve injury [142]. Therefore, reorganization of the brain during transition from acute to chronic pain has been demonstrated in animal pain models. These preclinical investigations are beginning to identify molecular underpinnings of the shift from nociceptive to emotional pain processing in chronic pain.

## 4. Translation of Preclinical Findings to Clinical Applications

Research in animals allows invasive mechanistic investigations that can be performed in a relatively homogenous population under calibrated and controlled conditions without the interference of confounding factors such as use of medications, different life styles, and so on present in human studies. Animal research has significantly contributed to our understanding of basic anatomy and neural mechanisms of pain processing. For example, the descending opioid-sensitive pain-modulatory pathways from the periaqueductal gray area (PAG) and rostral ventromedial medulla (RVM) were first identified in rodents and cats [143–145]. It was later discovered that electrical stimulation of PAG produced naloxone-reversible analgesia in patients with intractable pain [146, 147]. This success in predicting clinical responsiveness based on preclinical findings demonstrates translational relevance of animal research. However, limitations of animal research must also be acknowledged.

First, animal pain models cannot directly replicate the full complexity of the human pain experience and are intended to inform about potentially important neural mechanisms. Second, animal research is conducted in homogenous populations, offering an advantage in robustness of the effect size, but also potentially limiting the generality of the effect. Several examples have shown differences in pain responses between different species or even between different rodent strains [148, 149]. Another obstacle specifically in investigating chronification of pain in animals is the relative inability to assess pain-related emotional states. Researchers have used open-field, forced-swim, or elevated plus-maze tests to evaluate anxiety- and depressive-like behaviors in animals with persistent pain [150, 151]. Burrowing, home-cage monitoring, and voluntary wheel running have been used to assess the well-being of animals [152, 153]. Recently, operant assays such as conditioned place preference/avoidance [154] and place escape avoidance paradigm (PEAP) [155] were adopted to evaluate affective-motivational aspects of acute and ongoing pain. Although these measures are indirect and limited in comparison to the whole array of affective, emotional, and well-being assessments available in human research, they provide important mechanistic information about emotional-motivational and cognitive aspects of pain. An important issue for future research is thus to increase correlation between human and animal studies. Such complementary research is necessary to identify the cellular and molecular mechanisms that promote transition to chronic pain and determine whether these mechanisms can be targeted therapeutically.

## 5. Conclusions

Chronic pain is different from acute nociception or subacute persistent pain. Unrelenting pain loses its alerting and motivational utility and becomes a constant burden that disrupts goal-directed behavior. Patients with chronic pain are often more vigilant to pain-related information and shift their motivational priority to pain control goals. The affective and motivational disturbances play a critical role in reward, learning and information processing, goal regulation, and avoidance behavior. These observations suggest a complex interplay between motivational and emotional factors, whose interrelations and relevance shift in chronic pain. Related variables such as valuation of stimuli, desires, and needs vary largely inter- and intraindividually depending on a specific situation.

Consistent with these psychological signs of chronic pain patients, neuroimaging studies have demonstrated changes in brain anatomy and function, that generally reflect a shift from predominantly nociceptive to more affective and emotional processing [156]. Altered structure and function in emotional-motivational frontostriatal brain circuits have been shown to predict the transition from subacute to chronic back pain. Specifically, increased functional connectivity in between the nucleus accumbens and the ventromedial PFC predicted this transition [157] as well as higher incidence of white matter and functional connections between medial prefrontal cortex, NAcc, and amygdala [138, 158]. Both these functional and structural alterations predicted pain persistence over one year, suggesting that the development of chronic pain is predetermined by neuroanatomical and neurofunctional factors outside core nociceptive processes. Importantly, the relevance of nonnociceptive frontostriatal circuits in pain modulation has been confirmed in healthy volunteers [109, 159]. In addition, altered dopamine and opioid activity have been shown using PET imaging. Research in humans, however, is limited when it comes to the investigation of underlying neurophysiological mechanisms. Despite great advances in technical possibilities, brain imaging is still restricted to a coarse resolution compared to the density of neurons, and invasive procedures are only possible in very rare cases.

Animal research has confirmed the role of corticolimbic circuits in affective and motivational aspects of pain and provided more fundamental insights into neurobiological mechanisms. Thus, electrophysiological, FSCV, and behavioral measurements demonstrate a role of dopamine signaling for pain and pain relief. Other investigations show the requirement of endogenous opioid activity in the ACC for relief of pain following opioid or nonopioid therapy. With the development of new techniques, including genetic approaches, it is now possible to investigate the impact of chronic pain on specific cells in neuronal circuits. Translationally relevant animal models and measures that are based on clinical observations will be able to provide mechanistic insights into neural circuitry in chronic pain and help to identify novel therapeutic options for patients.
